# Transforming Professionalism Education in Clerkships: A Student-Driven Approach Utilizing The Hidden Curriculum

**DOI:** 10.5334/pme.1572

**Published:** 2025-04-07

**Authors:** Munawar Farooq, Azhar T. Rahma, Zufishan Alam, Mohammad Al Banna, Uffaira Hafeez, David O. Alao, Arif Alper Cevik

**Affiliations:** 1 Department of Internal Medicine, Emergency Medicine Section, College of Medicine and Health Sciences, United Arab Emirates University (UAEU), Al Ain, AE; 2 Emergency Department, Tawam Hospital Al Ain, AE; 3 Institute of Public Health, College of Medicine and Health Sciences, UAEU, Al Ain, AE; 4 School of Health and Environmental Studies, Hamdan Bin Mohammed Smart University, Dubai, AE; 5 Mohammed Bin Rashid University of Medicine and Health Sciences, Dubai, AE; 6 Department of Internal Medicine, Emergency Medicine Section, College of Medicine and Health Sciences, UAEU, Al Ain, AE; 7 Emergency Department, Tawam Hospital Al Ain, AE

## Abstract

**Introduction:**

Professionalism, influenced by regional context and societal values, is essential in doctor-patient relationships, patient experiences, and clinical outcomes. However, formal education alone fails to cultivate professionalism effectively. Research highlights the hidden curriculum’s detrimental impact on medical students’ professionalism. Nonetheless, strategies to teach professionalism in specific curriculum areas and to counteract hidden curricula, particularly for clinical clerkships, remain underexplored. This study evaluates a structured, student-led professionalism training program in a clerkship.

**Methods:**

Over one year, we implemented and replicated an educational intervention on professionalism in four emergency medicine clerkship groups. Grounded in constructivist and transformative learning theories, the intervention aimed to enhance students’ reflective capacities by addressing the hidden curriculum. It included briefing sessions on professionalism models and student-led discussions on clinical cases encountered to uncover implicit lessons. Students’ understanding was reinforced through anonymous self- and peer assessments of professionalism traits. The impact was evaluated qualitatively through inductive thematic analysis of student reflections and quantitatively through student feedback based on the Kirkpatrick model.

**Results:**

The training received highly positive evaluations from students. Quantitative analysis showed significant score increases in knowledge and ability (using the Wilcoxon Signed Rank Test). Students demonstrated the ability to reflect on the hidden curriculum and highlighted three key themes: professional attributes, sociocultural context, and system-level factors. Subthemes included communication, empathy, learning commitment, cultural competence, hierarchy, and family engagement.

**Discussion:**

This study presents a practical clerkship professionalism training model demonstrating that regular case-based discussions and anonymous self- and peer assessments help students identify and reflect on professional behaviors within the hidden curriculum.

## Introduction

Professionalism is an evolving construct influenced by regional context, including existing values and beliefs of a society [1]. While there have been many attempts to define professionalism, no standardized or universally agreed-upon definition exists. The Royal College of Physicians defines medical professionalism as “a set of values, behaviors, and relationships that underpins the public’s trust in doctors [2].” The American Board of Internal Medicine identifies six key dimensions of professionalism: altruism, accountability, excellence, duty, honor and integrity, and respect for others, and also highlights five threats to professionalism: abuse of power and harassment, conflicts of interest, professional arrogance, physician impairment, and research fraud [3]. Medical professionalism impacts doctor-patient relationships, patient experience, and clinical outcomes [4]. Professionalism is a fundamental competency across various medical education frameworks [5].

Several studies reported that medical students’ moral and ethical reasoning declines throughout their medical school studies, especially through their clinical years of training [6]. This decline may be attributed to the hidden curriculum, a term that refers to clinicians learning the aspects of their profession through real-world experiences in the clinical setting, often through informal or unintended education [7]. Students acquire positive and negative traits by absorbing these implicit lessons from their day-to-day observations and interactions. The conflict between what is formally taught and what is observed from superiors gradually erodes professionalism and normalizes unprofessional behavior [8]. Joynt et al. found a significant disparity between the knowledge imparted by the formal curriculum and the perceptions shaped by the hidden curriculum [9]. Multiple other factors can contribute to unprofessional behavior, such as deficiencies in the educational system, cultural and social dynamics, and the personal struggles of students [10].

Conventionally, medical professionalism is taught through various methods, including didactic lectures, web-based modules, and role modeling [11]. However, formal education fails to instill professional behaviors in medical students consistently. Experts recommend leveraging the hidden curriculum’s significant influence to foster professionalism in students, recognizing its crucial role in shaping future clinicians’ values and behaviors [12]. Effective training nurtures professionalism by transforming institutional culture and mitigating the hidden curriculum’s impact [13].

Professionalism training in medical education often utilizes dedicated sessions offered separately or as part of a longitudinal design [14,15]. However, practical guidance for integrating professionalism training into individual courses or clerkships while counteracting hidden curricula is limited. Teaching professionalism in time-limited clinical clerkships is challenging, often overshadowed by immediate clinical priorities, and remains under-researched. Our study addresses this gap by implementing and evaluating structured, student-led professionalism training in emergency medicine clerkships grounded in well-recognized learning theories [16–18]. This approach unpacks the hidden curriculum and enhances reflective practice, offering an adaptable model for various courses and clerkships.

The study aimed to implement and evaluate a structured, student-led approach to learning professionalism within the Emergency Medicine Clerkship, focusing on addressing and unpacking the hidden curriculum.

## Methods

### Study design and settings

This prospective educational intervention used a mixed-method approach in the Emergency Medicine Clerkship at UAEU’s College of Medicine and Health Sciences. At the time, no formal professionalism training was offered after students’ first two years. The study period spanned from September 2023 to August 2024.

### Participants

Sixty-eight final-year students participated in a four-week Emergency Medicine Clerkship, organized into four groups of 16–18 students. Each group was further divided into subgroups of 5–6 students. Teaching methods and professionalism assessments were standardized and applied consistently across all groups throughout the academic year.

### The training intervention

The main strategies for promoting professionalism included student-led discussions, reflective activities, and examining the hidden curriculum during clinical encounters, guided by constructivist and transformative learning theories.

The constructivist theory emphasizes active learning, where students construct knowledge through engagement, building on prior knowledge by connecting new information to existing ideas, and social and cultural learning, which enhances understanding through collaboration within a social context [16,17]. Transformative learning theory emphasizes critical reflection, challenging assumptions to deepen understanding; reflective discourse, using dialogue to explore diverse perspectives; and identity transformation, where new experiences reshape one’s worldview and self-concept [18].

### Intervention design and implementation

Collaborating with colleagues, a clerkship faculty member interested in professionalism and human factors integrated professionalism training into the Emergency Medicine Clerkship curriculum through a student-centered intervention. Aligned with program learning outcomes, the intervention aimed to develop professionalism competence by fostering commitment to professional responsibilities and ethical principles. Students were encouraged to observe aspects of professionalism in clinical settings and share their reflections, shaping the training content based on their experiences rather than being taught a predetermined set of professionalism topics.

#### Orientation meeting

Using simplified models, students learned core professionalism principles [19]. The clerkship activities were signposted as learning experiences with clear expectations for professional behavior. Students were introduced to the concepts of intended and hidden curricula and were encouraged to observe and reflect on professionalism in clinical encounters [7]. They were asked to present their observations in twice weekly morning meetings, engaging in group discussions facilitated by faculty, who provided guidance and positive reinforcement. In the third week, students could submit an optional unstructured reflection on professionalism issues. While participation in discussions was a mandatory part of the clerkship, the written reflection was optional.

#### Morning meetings

Twice-weekly discussions centered on students’ lead clinical reflections. These sessions promoted collaborative analysis of ethical dilemmas, enhancing knowledge through peer interaction. For example, students shared observations of patients requesting narcotic analgesia, noting varied team responses to highlight the interplay of active listening, self-control, empathy, patient-centered care, accountability, integrity, and system-based practice in such presentations.

#### Self and peer assessments

During the first week, students completed anonymous self-assessments of professionalism using the professionalism assessment scale via an online survey [20]. In the final week, each student conducted anonymous peer assessments of five subgroup members using the same scale previously used for self-assessment. This process fostered self-reflection, accountability, and tolerance for constructive criticism. Scores were visible only to the assessed students and were not collected or published. Emphasizing its role as a learning tool rather than an evaluative measure, the anonymity of the process was ensured to encourage honest and constructive feedback.

#### Workshop on professionalism and human factors

The clerkship included a faculty-led 90-minute workshop on human factors, incorporating a discussion on professionalism. To prepare for the workshop, students were provided with two pre-reading articles on professionalism in medical education and human factors in emergency medicine [19,21]. Scenarios from emergency medicine practice and literature prompted students to assess and justify the professionalism of specific medical student behaviors [22].

#### Reflective assignments

Students analyzed their experiences and understanding of professionalism through written reflective assignments, identifying discrepancies between the clerkship’s intended curriculum (expected behaviors) and the hidden curriculum (observed behaviors). Group discussions during morning meetings, as stated above, prepared students for this written reflection. Students submitted reflective writings on professionalism in a clinical case from their placement without specific structural or word count requirements. This process refined their professional perspectives and enabled faculty to appreciate students’ abilities to recognize and evaluate professional behaviors.

**Figure 1:**
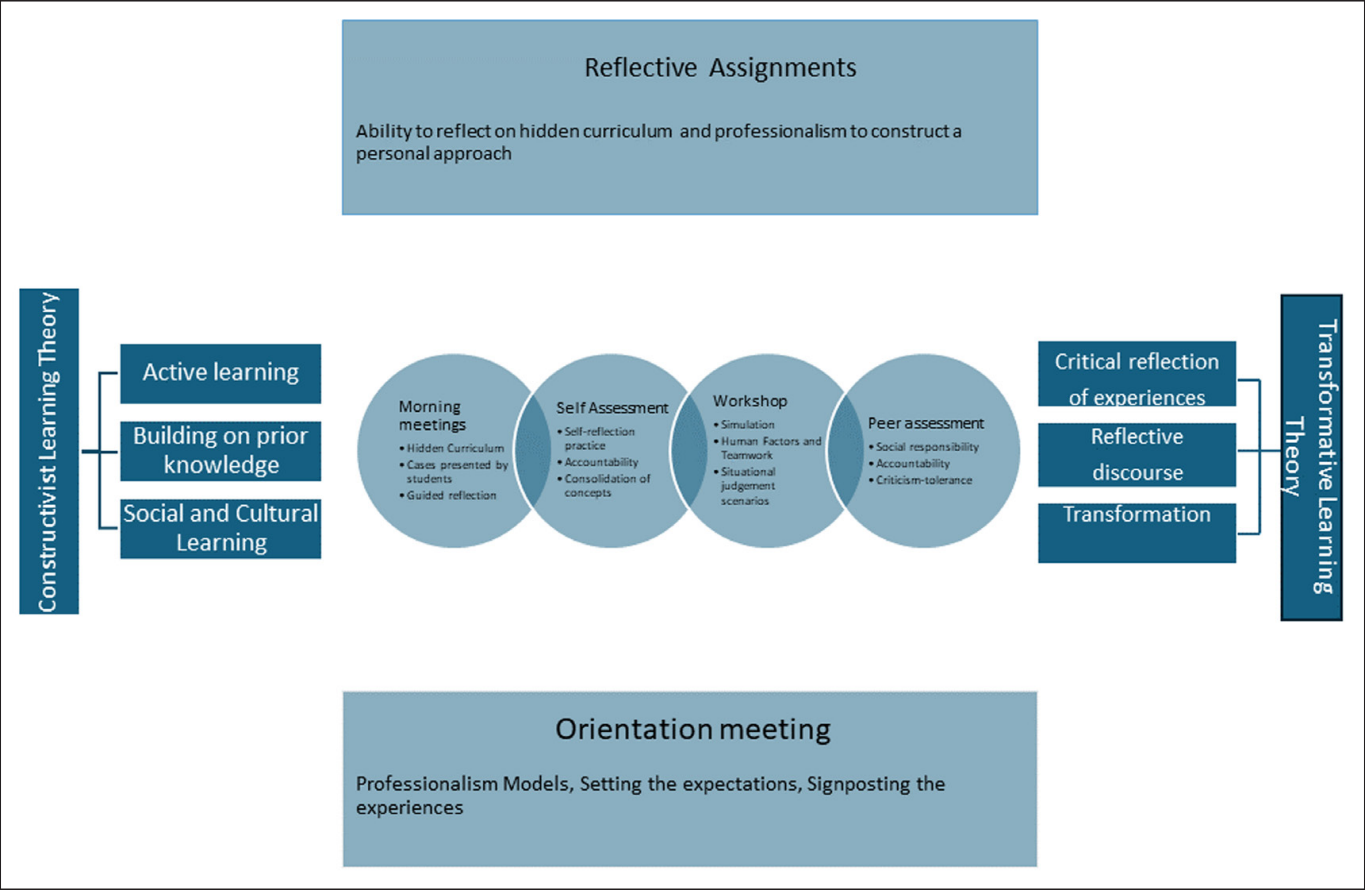
Professionalism Training in Clerkship Through Hidden Curriculum Insights.

These components aimed to deepen student engagement with professionalism, integrating constructivist and transformative learning theories to promote active learning, reflection, and personal growth. (Figure 1).

### Assessment of Educational Intervention

The intervention’s effectiveness was assessed through qualitative and quantitative evaluations.

#### Quantitative Evaluation

The quantitative evaluation used the Kirkpatrick model [23] to gather feedback on participants’ satisfaction and training perceptions. Students were asked to rate their knowledge and ability regarding professionalism. The faculty decided on the categories used in the questionnaire for quantitative analysis based on professionalism models shared with students in the orientation meeting and the pre-reading for the professionalism workshop. Knowledge was assessed across six aspects, each scored on a 5-point scale, with a maximum total score of 30. Ability was assessed through three aspects, each scored on a 5-point scale, with a maximum total score of 15. Individual knowledge and ability scores were summed for each student to generate total scores before and after the intervention. Since the data were ordinal, the Wilcoxon signed-rank test was applied to rank differences. Data were analyzed with SPSS (v29). Evaluation questions are provided in Appendix A.

#### Qualitative Evaluation

Reflective assignments were qualitatively evaluated to assess students’ abilities to observe and analyze professionalism issues in clinical practice, indicating students’ awareness and competency regarding professionalism. The qualitative data from reflective assignments submitted by medical students were analyzed using an inductive thematic analysis to identify themes [24]. The cases were encoded, themes were recognized, and inter-coder reliability was validated. Codes were refined iteratively to ensure clarity and robustness and then grouped into cohesive and overarching themes. Data were uploaded for analysis, and results were displayed using NVivo 12 (Windows version) [25] and the Artificial Intelligence tool (Avidnote) [26].

We employed ongoing discussions throughout the coding and analysis process to ensure reflexivity, critically examining our assumptions and biases on theme development. Additionally, AI-supported secondary validation using Avidnote helped us minimize subjectivity. Avidnote tool supported the thematic analysis. After uploading the data, we used Avidnote to highlight key quotations, categorize information, and create meaningful codes. The tool’s ability to group related codes helped us refine our themes. By allowing side-by-side comparisons and iterative adjustments, Avidnote acted as an extra check, complementing the work of two independent researchers (A.T.R & Z.A.) to enhance inter-coder reliability. The themes were visualized using the Xmind app [27]. The findings are reported using the consolidated criteria for reporting qualitative research (COREQ) checklists [28].

### Ethics Approval

The UAEU Social Sciences Ethics Committee approved the study – with Approval No: ERSC_2023_3652. All medical students provided informed consent for voluntary participation in the research and the publication of the study results.

## Results

### Quantitative Findings

**Figure 2:**
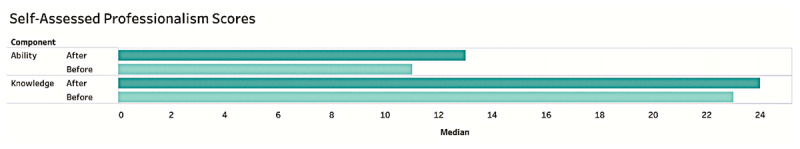
Self-assessed professionalism scores.

Responses were received from 53 participants, resulting in 53 pairs for the quantitative analysis. The average age of the students was 22.8 ± 1.02 years, with 78% of them being female. The median post-intervention professionalism scores for knowledge and ability were significantly higher than pre-intervention scores (Figure 2).

The Wilcoxon Signed Rank Test revealed a statistically significant increase in scores, with Z = –4.275, p < .001 for knowledge and Z = –4.002, p < .001 for ability.

We received excellent feedback, which was collected on the amount learned, content, and class time, using a 1–10 scale where one represented “None/Not Good” and 10 represented “A Lot/Great.” The median ratings were 9 for the amount learned, 10 for the content, and 9 for the use of class time.

### Qualitative Findings

The qualitative analysis included all thirty-one student reflections on professionalism issues in various clinical encounters. The clinical encounters included both male and female patients, with an age range of 3–79 years. The student reflections were based on a wide range of case presentations, from pain management due to various reasons to cardiovascular incidents, altered mental status, and death. The patients either attended alone, were accompanied by friends and relatives, or were brought by ambulance. The reflected encounters included consultants, doctors, nurses, Patient Relations Officers (PROs), and medical students.

**Figure 3:**
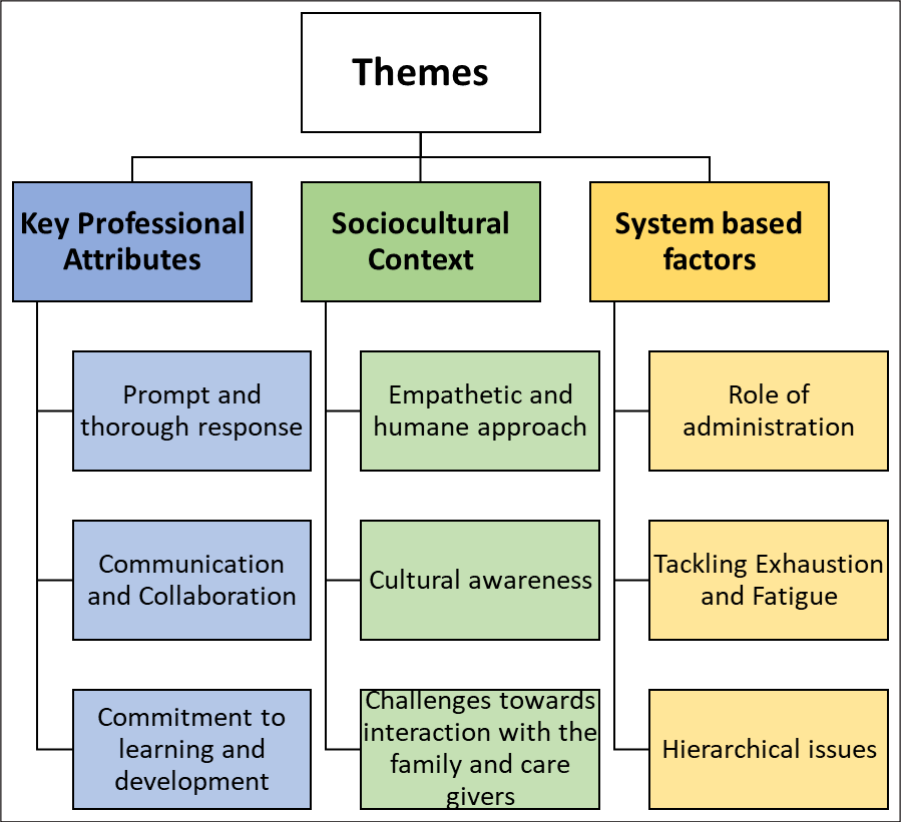
Visual representation of main themes and subthemes extracted based on inductive thematic analysis of students’ refelctions.

The analysis revealed that behaviors seen in clinical practice (the hidden curriculum) often differ from the formal standards taught in medical school. This is supported by the identified themes and sub-themes, which include key professional attributes, sociocultural context, and system-based factors. Figure 3 illustrates these themes and their corresponding sub-themes. Students reflected on both positive and negative examples of professionalism within each sub-theme.

#### 1. Key Professional Attributes

These attributes refer to the essential behaviors, skills, and attitudes that define professionalism in emergency physicians. Students analyzed instances of positive and negative behaviors, including prompt and thorough response, effective communication and collaboration, and commitment to learning and development.

##### 1.1 Prompt and thorough response

Emergency room physicians must respond promptly and thoroughly to patient needs, overcoming personal or situational biases. Students observed the importance of timely patient management, emphasizing history-taking, rapid exams, and urgent tests. They noted that deficiencies, such as over-reliance on lab tests, can lead to missed diagnoses and inadequate care.

‘No need. I have already ordered the tests. Let’s wait and see what the results show (BC5).’

Students identified the importance of recognizing patient distress, calling for assistance, and swiftly initiating treatment and intervention. Students also outlined appropriate steps for patient monitoring and handover, emphasizing that neglecting proper handover is unprofessional and unsafe for patient well-being, as reflected by one of the students:

‘The doctor might not have informed the team about the high risk of another seizure for the patient, and there was no designated plan for monitoring the patient to detect potential seizures promptly (BA5-2).’

##### 1.2 Effective communication and collaboration

The students keenly observed the effectiveness of communication and teamwork in the conduct of their seniors and elaborated on it in their reflections:

‘During that discussion, the patient appeared annoyed and frustrated with the doctor. However, the doctor tried to continue and recommended other pain management options that might be effective for his condition. Throughout the whole event, I was focused on how the doctor tried to deal with that condition; he showed excellent communication skills, starting from trying to figure out the main problem to avoid any misunderstanding or inappropriate judgment. (CA5-2).’

Students identified how poor communication about management plans frustrates patients, causing mistrust.

‘The doctor didn’t address the patient’s concerns about scarring. The patient likely didn’t understand the full treatment plan or the limitations on the plastic surgeon’s availability (BB4-3)’

The students analyzed how fragmented team approaches delay problem identification and patient care, unlike cases where effective collaboration was demonstrated.

‘Collaborative teamwork among the doctors, nurses, and residents is essential in such cases to prevent confusion among the team. Having multiple people visit the patient at different times and report different findings can make formulating an effective treatment plan challenging (CA3-1).’

They concluded that proper division and designation of responsibilities among members, including themselves, would be fruitful, as everyone’s expertise would be utilized.

##### 1.3 Commitment to learning and development

Another professional attribute that students frequently reflected upon was the zeal for continuous learning. Providing opportunities to learn from one’s mistakes was perceived as a significant facilitator for instigating professionalism in one’s behavior.

‘The consultant and the resident admitted their mistake of not noticing earlier and immediately started rereading the ECGs more thoroughly to provide the best care for the patient. The consultant doctor decided that it was important to do a quick teaching session for me and the other residents present that day about step-wise ECG reading. Seeing a mistake being noticed quickly and having a team who is confident enough to admit their mistake and fix it immediately was a good learning point from this shift (BB6-1)’

#### 2. Sociocultural Context

Students reflected upon the connection between professionalism and sociocultural aspects of a health professional’s behavior based on the following sub-themes:

##### 2.1 Empathetic and humane approach

Students frequently acknowledged the positive effect of attending practitioners’ compassionate behavior on patients. They signified that taking time to explain to the patient, offering an apology when needed, reassuring them about their condition, considering them humans, and giving importance to their feelings were pivotal to a professional attitude.

‘I like that the doctor was putting the patient as a whole at the center of care, trying to address his issue and pain in the appropriate way while taking into consideration and prioritizing his overall health and minimizing the risks. He tried to educate the patient regarding his current situation in simple words to involve him in the decision-making process (CA4).’

‘The doctor was talking to him and reassuring him that everything would be fine. The doctor held his hand and spent time talking to him, trying to console him, and offering reassurance (BA2-2).’

Students acknowledged that respecting patient autonomy and obtaining consent are essential to their conduct, enabling them to identify violations of consent or confidentiality.

‘Meanwhile, the medical student conducting the physical examination encountered a critical mistake. The student failed to obtain proper consent before exposing the patient’s abdominal area and unintentionally compromised the examination area’s privacy by not closing the opened curtain. The patient felt upset, quickly stopped the exam, and asked the student to leave (CA5).’

Students understood the need to accommodate patient preferences without being judgemental. However, they also expressed that some patient decisions might not be the best for their interest, where professionals should intervene. For instance, when a patient suffering from dizziness walked away by himself and signed for leave against medical advice (LAMA), he had a car accident on his way home. The scenario was interpreted by the student as follows:

‘The patient leaving against medical advice (LAMA) and experiencing a car accident highlights the potential challenges in maintaining continuity of care and ensuring patient safety. In this context, human factors, including patient education, follow-up care, and the patient’s decision-making process, become crucial elements in preventing adverse outcomes (BC2).’

##### 2.2 Cultural awareness

Students identified cultural responsiveness as being crucial for professional practice in emergency healthcare. They described challenges in communicating with patients from diverse backgrounds and suggested strategies to overcome such barriers. Discriminatory practices were identified and strongly discouraged, with students acknowledging that racial bias could impair the quality of care.

‘So, while we were heading toward the patient, the doctor read the patient’s nationality, so he decided to skip the patient and return later because he thought that people from this country exaggerated the symptoms and just needed a sick leave. This situation annoyed me because every patient has the right to be listened to carefully to never miss any red flags and take a proper history and physical examination and never judge the person based on the nationality background (CC7).’

According to the students, understanding stigma related to specific medical issues is crucial, and potential cultural insensitivity could lead to conflict. For instance, when a pregnancy test for investigation of abdominal pain was ordered for a female single patient, it led to a misunderstanding, with the patient being furious and dissatisfied over the societal implication associated with consideration of pregnancy out of marriage in the local culture:

‘The patient started to scream and felt angry for that as they were requesting a pregnancy test while they knew that she was single. For me, I agree with the doctor that we always need to investigate for everything to avoid missing anything that would threaten the patient’s life. However, I think that it is known that this is considered unacceptable in our society, especially among the locals. So, I prefer not to mention specifically the types of tests (pregnancy test specifically) that were ordered to avoid any problems or misunderstanding such as in this case (CA4-1).’

##### 2.3 Challenges toward interaction with the family and patient carers

Students reflected on the importance of engaging professionally with patients’ family members, noting that this role is as critical as interacting with the patient. They observed that family members often struggle with receiving bad news or dealing with grief. They emphasized the need for sympathetic and appropriate responses in such situations. For example, when a distressed relative was informed about his mother’s condition, the attending doctor demonstrated professionalism by prioritizing both the patient’s well-being and the emotional needs of the relative, offering support in a quiet area. In contrast, students also reflected on times when necessary empathy was not displayed while breaking bad news:

‘Unfortunately, regardless of their enormous effort, the patient was declared dead, and the mother was poorly dealt with; there was no empathy in breaking bad news to her (CB3).’

In some instances, students observed that family accompanying patients was the best source of information regarding the patient’s condition. In contrast, sometimes family members were seen to withhold essential details required to guide toward proper diagnosis. Students reflected that this could happen when family members were afraid to disclose information that may lead to stigma associated with particular issues such as suicide or alcohol use.

‘Initially, the family withheld information about the patient’s alcohol consumption, attributing his condition to a potential issue with his blood sugar levels (CB1).’

Thus, students comprehended the importance of fostering open communication and trust between healthcare providers, patients, and their families to prevent delayed diagnosis and management of the issue.

#### 3. System based factors

System-based factors in the Emergency Room (ER) refer to the structural, administrative, and organizational pillars that influence care delivery. The students reflected that these factors are essential in shaping professionalism, teamwork, and patient safety. The student reflections identified several system-level factors interlinked with professionalism, including the administration’s role, physician fatigue and exhaustion, and hierarchical issues.

##### 3.1 Role of administration

Students comprehended the administration’s role in emergencies, specifically that of the patient relations officer (PRO). According to the students, PROs played an important role in the de-escalatory efforts in certain situations, such as when facing aggressive behaviors by patients and their carers. In one instance, when a son of the patient became angry, the PRO intervened to address the patient’s frustration and attempted to clarify the situation,

‘He got in a fight with the security. He tried to open the resuscitation door, until he did. He distracted the team in the resuscitation area. He shifted their attention from the patients to the disruption he made. PRO came to handle the situation (BC3-2)’

Students observed police involvement in critical trauma situations and acknowledged the medical team’s role in aiding investigations for law and order. This was illustrated when a student facilitated communication between the police and a patient, ensuring clarity and accuracy of information.

‘This is a police investigation, and anything being said cannot be taken lightly. Delivering information in certain words or styles could give a different meaning and hold different consequences. I felt responsible, and I wanted to do well in this (CC2-1).’

##### 3.2 Tackling Exhaustion and Fatigue

One of the challenges that students reflected upon in their practice of assigned roles in the Emergency Department was long shift hours with little or no break in between, which led to fatigue. Students reflected that the weariness and burnout may lead to cognitive impairment, impact clinical performance, and affect healthcare provision, thus compromising patient safety:

‘I began wondering how emergency medicine physicians deal with fatigue, especially as they are required to deal with life-threatening cases at any time during their shift. The prolonged work hours may negatively impact cognitive performance and hinder decision-making, especially toward the end of a shift. The risk of medical errors, misdiagnoses, and suboptimal patient care increases when healthcare providers are fatigued. Patient safety becomes a major concern (CA1).’

They stressed the importance of adequate breaks and fair division of responsibility among team members.

‘A solution to this problem can be implementing fatigue management strategies and optimizing physicians’ work hours to include rest breaks and the ability to regroup tasks to ensure adequate rest during the shift (CA1).’

##### 3.3 Hierarchical issues

Students shared challenging interactions with seniors on a medical team, sometimes feeling unsupported by their peers. They found that power dynamics prevented them from refusing tasks they felt unqualified for or questioning the senior residents. For instance, one student hesitated to voice concerns about suturing, fearing she would seem incompetent before a senior doctor:

‘I started to feel a little bit nervous because I did not know what was expected of me, and I was scared that maybe I would do something wrong; however, I decided to remain silent because I did not want to lose this opportunity. I would not want to lie; I was feeling a little scared and felt like I was doing something wrong. I had a lot of choices to choose from moving forward, but I chose to stay quiet and do exactly what she said; in the process, I poked my finger with the needle. I started panicking a bit because I would not be able to tell anyone outside the room I was in because it would cause many problems for the resident (CB4).’

Students advocated for a system where structural balance exists between junior and senior members of the medical team, allowing open communication without any apprehension or anxiety when voicing worries or asking for assistance:

‘The assembly of manpower and task division in a busy ER where each tasked individual is given a role that fits the task level of urgency and their individual level in the hierarchy of a medical team reflects the wisdom of employing human factors to maximize patient care and situation management (CC2-1).’

## Discussion

This study offers an effective model for teaching professionalism in time-limited courses and clerkships based on constructivism and transformative learning theories. The orientation meetings and morning discussions fostered social learning by engaging students in dialogues about professionalism and ethical behavior. Transformative learning theory, focused on critical reflection and perspective transformation, was incorporated through self and peer assessments, interactive workshop activities, and structured reflective practice exercises. These exercises encouraged students to explore the professional attributes, sociocultural aspects of professionalism, empathy, and system-level factors in their reflections, allowing them to critically analyze their own and their peers’ actions and observations. The analysis reveals that observed behaviors in clinical practice (the hidden curriculum) often diverge from the standards taught in medical school. The training received highly positive evaluations from students, who reported substantial improvements in knowledge and reflective capacity in professionalism.

Longitudinal integrated clerkships have demonstrated efficacy in fostering professional identity development; however, implementing structured professionalism training within existing 4 – to 8-week courses or clerkships continues to pose significant challenges [29]. These challenges are particularly evident as students confront ethical dilemmas and inconsistencies during their clinical experiences [30]. In this investigation, the proposed model for professionalism training within the context of a clinical clerkship was successfully replicated across four distinct student cohorts as they rotated through Emergency medicine clerkships in their final year.

The themes identified from student reflections closely align with established standards of medical professionalism. Students underscored the importance of effective communication in optimal patient management, reinforcing literature that advocates for early training in communication skills as a fundamental component of professionalism for undergraduate students [31]. They also emphasized teamwork for seamless patient care, enhancing clinical outcomes [32].

Students appreciated the doctors’ commitment to continuous learning, linked to increased professional effectiveness [33]. Additionally, students reflected on the significance of understanding cultural contexts and perceptions in medical practice. Cultural competency and humility are core elements of patient-centered care, particularly in a diverse population such as the UAE [34]. Furthermore, students recognized the impact of system-level factors on doctors’ professionalism, highlighting the influence of administrative roles. Evidence suggests that institutional culture and behavior significantly shape physicians’ professional conduct [35]. Students also expressed concern about the adverse effects of long shifts and burnout on clinical performance and patient safety. Substantial evidence indicates that burnout compromises patient safety and physicians’ professionalism [36].

The results of our study underscore the importance of addressing the critical role of the hidden curriculum in shaping professional behavior. In a study, students highlighted that care was often more disease-oriented, with communication and aspects of the doctor-patient relationship frequently neglected [37]. Another study from Harvard Medical School explored the factors that influence the changes students observe in themselves during medical training. The hidden curriculum emerged as a key theme, including behavioral modeling and values such as compassion, patient-centered care, teamwork, and communication [38]. In another study, undergraduate and postgraduate students noted a culture tolerant of unprofessional behavior [39]. Mackin et al.studied the lived experiences of healthcare professionals regarding the hidden curriculum; participants expressed a disconnect between what they are taught and what they encounter in practice [40]. In another study, students were given sessions on the hidden curriculum, and they could subsequently recognize positive and negative experiences [41]. The intervention proposed in this study deliberately utilizes the hidden curriculum to promote competency of reflection and professionalism.

Incorporating reflective writing helps students comprehend and reflect upon their clinical experiences and mistakes [42]. Students identify interactions that do not align with the standards of professionalism. One study builds on the evidence that the hidden curriculum in medical training impacts students’ decisions to raise concerns when they observe unprofessional behavior [43]. Additionally, as our students pointed out, we recommend addressing system-level factors contributing to accepting unprofessional behavior. Martimianakis et al. found that most programs pay minimal attention to the organizational role in propagating unprofessional behaviors [44]. Addressing these factors at the organizational level is crucial for fostering a professional and ethical learning environment.

### Limitations

This single-center study, conducted over a 4-week EM clerkship, may limit the generalizability of its findings. However, the teaching model was applied consistently across four student groups, demonstrating reproducibility. Additionally, the qualitative analysis relied on reflective essays, which, while valuable, may not have fully captured all dimensions of professionalism. Another limitation of this study is the voluntary nature of reflective writing and feedback completion, which may have introduced selection bias. Of 68 students, only 31 provided reflective writing, while 58 completed pre- and post-study self-assessments. This self-selection could limit the generalizability of the findings, as more motivated students may have been overrepresented. Furthermore, the analysis relied on self-reported improvements rather than 360-degree assessments. In longer clerkships or residency programs, yearly reflections coupled with 360-degree assessments would be more valid, but these are not feasible for short-duration clerkships.

The study noted reports of unprofessional and unsafe behaviors in reflections, but no formal reports were made due to anonymity. General feedback was provided to ED leadership to improve the clinical learning environment.

## Conclusion

This study introduces an effective clerkship professionalism training model that enables students to reflect on professional behaviors within the hidden curriculum. Written reflections highlighted students’ recognition of professional attributes, sociocultural factors, and system-level influences, with sensitivity to communication, empathy, and patient-centered care. The intervention’s core principles—reflection, constructivism, and critical analysis of the hidden curriculum—are adaptable to various specialties and clinical contexts, offering a transferable framework for enhancing professionalism training globally.

## Data Availability

The data that support the findings of this study are available from the corresponding author (MF), upon reasonable request.

## Appendix A: Questions for Quantitative Evaluation

### Knowledge

**Grade your knowledge/understanding on the following professionalism aspects:**

1. What is Medical Professionalism?
2. Integrity in professionals.
3. Involvement and professionalism in medical education and practice.
4. Professional interactions.
5. Value of introspection for medical students.
6. Local context and culture in professionalism.

### Ability

**Grade your ability in the practical application of the following professionalism aspects:**

1. Ability to identify professionalism aspects in medical education and practice.
2. Ability to reflect and analyze professional behaviors.
3. Ability to develop a personal approach toward professional behavior in medical education and practice.

### Feedback

1. On a scale of 1–10, 1 = NONE/NOT GOOD and 10 = A LOT/GREAT. Circle a number for THE AMOUNT YOU LEARNED about professionalism.
2. On a scale of 1–10, 1 = NONE/NOT GOOD and 10 = A LOT/GREAT. Circle a number to indicate how useful THE CONTENT presented in this clerkship was.
3. On a scale of 1–10, 1 = NONE/NOT GOOD and 10 = A LOT/GREAT. Circle a number for THE USE OF CLASS TIME for professionalism learning.
